# IL-6 inhibition with clazakizumab in patients receiving maintenance dialysis: a randomized phase 2b trial

**DOI:** 10.1038/s41591-024-03043-1

**Published:** 2024-05-25

**Authors:** Glenn M. Chertow, Anna Marie Chang, G. Michael Felker, Mark Heise, Elena Velkoska, Bengt Fellström, David M. Charytan, Regina Clementi, C. Michael Gibson, Shaun G. Goodman, Meg Jardine, Adeera Levin, Yuliya Lokhnygina, Jenny Mears, Roxana Mehran, Peter Stenvinkel, Angela Yee-Moon Wang, David C. Wheeler, Carmine Zoccali, Paul M. Ridker, Kenneth W. Mahaffey, Pierluigi Tricoci, Myles Wolf

**Affiliations:** 1 Stanford University, Palo Alto, CA USA; 2 CSL Behring, King of Prussia, PA USA; 3 Duke University, Durham, NC USA; 4 CSL Limited, Melbourne, Victoria Australia; 5 Uppsala University, Uppsala, Sweden; 6 New York University, New York City, NY USA; 7 Harvard Medical School, Boston, MA USA; 8 University of Toronto and University of Alberta, Edmonton, Alberta Canada; 9 University of Sydney, Sydney, New South Wales Australia; 10 University of British Columbia, Vancouver, British Columbia Canada; 11 Mt Sinai Hospital, New York City, NY USA; 12 Karolinska Institute, Solna, Sweden; 13 Hong Kong University, Hong Kong, China; 14 University College London, London, UK; 15 Ospedali Riuniti Reggio Calabria, Reggio di Calabria, Italy

**Keywords:** Vascular diseases, Kidney diseases

## Abstract

Inflammation mediated by interleukin-6 (IL-6) is strongly associated with cardiovascular risk. Here we evaluated clazakizumab, a monoclonal antibody targeting the IL-6 ligand, in a phase 2b dose-finding study. Adults with cardiovascular disease and/or diabetes receiving maintenance dialysis with high-sensitivity C-reactive protein (hs-CRP) ≥ 2 mg l^−1^ at baseline were randomized to receive clazakizumab (2.5 mg, 5 mg or 10 mg, *n* = 32 per dose group) or placebo (*n* = 31) every 4 weeks. The primary endpoint was the change from baseline in hs-CRP to week 12, expressed as the geometric mean ratio. Clazakizumab treatment signficantly reduced serum hs-CRP concentrations at week 12 by 86%, 90% and 92% relative to placebo in patients randomized to 2.5 mg, 5 mg or 10 mg clazakizumab, respectively (all *P* < 0.0001), meeting the primary outcome. With regard to secondary endpoints, clazakizumab treatment reduced serum fibrinogen, amyloid A, secretory phospholipase A2, and lipoprotein(a) concentrations, as well as increased mean serum albumin concentrations at 12 weeks, relative to placebo. The proportion of patients who achieved hs-CRP < 2.0 mg l^−1^ was 79%, 82% and 79% in the 2.5 mg, 5 mg and 10 mg clazakizumab groups, respectively, compared with 0% of placebo-treated patients. With regard to safety, no cases of sustained grade 3 or 4 thrombocytopenia or neutropenia were observed. Serious infections were seen with similar frequency in the placebo, clazakizumab 2.5 mg and clazakizumab 5 mg groups, but were numerically more frequent in the clazakizumab 10 mg group. The results of this trial indicate that in patients receiving maintenance dialysis, clazakizumab reduced inflammatory biomarkers associated with cardiovascular events. ClinicalTrials.gov registration: NCT05485961.

## Abstract (web summary)

In a randomized clinical trial, treatment using a monoclonal antibody targeting interleukin-6 (IL-6) in patients with cardiovascular disease and/or diabetes who were receiving maintenance kidney dialysis reduced levels of inflammatory biomarkers at 12 weeks, including C-reactive protein, paving the way for a phase 3 trial.

## Main

Patients with end-stage kidney disease (ESKD) experience unacceptably high rates of mortality and morbidity despite the provision of dialysis. Average mortality rates adjusted for age, sex and designated race or ethnicity exceeded 19% in 2021; more than half of deaths were attributable to cardiovascular causes. Hospitalization and emergency department visit rates are also frequent, with roughly one-third attributable to cardiovascular disease^1^. ESKD and cardiovascular disease share an array of risk factors, including diabetes, hypertension, dyslipidemia and albuminuria, but cardiovascular event and death rates far exceed those expected^2^, suggesting that kidney disease-specific factors contribute to cardiovascular risk. Patients with ESKD have been excluded from most cardiovascular outcome trials although it is noteworthy that several large-scale trials of lipid-lowering agents in patients receiving dialysis failed to demonstrate a substantial benefit^3–5^.

In the general population, systemic inflammation is a major contributor to cardiovascular risk^6–9^. Evidence suggests a causal role of activation of the interleukin-6 (IL-6) pathway^10,11^. The Canakinumab Anti-inflammatory Thrombosis Outcomes Study (CANTOS) demonstrated that targeted use of IL-1β inhibition substantially reduced cardiovascular events^12^, particularly among patients with the largest downstream reduction in IL-6 (ref. ^13^). Chronic inflammation has also been implicated as a mediator of cardiovascular disease among patients receiving dialysis^14–16^. Many of the clinical manifestations of uremia—including pericarditis, pleuritis and serositis—are the result of visceral inflammation, and accelerated atherosclerosis in patients with kidney failure was recognized a half century ago^17^. Inflammatory markers, including serum concentrations of IL-6 and C-reactive protein (CRP), are typically elevated in ESKD and are associated with mortality and cardiovascular events in this population^18,19^. As such, it is plausible that a therapeutic approach targeting the IL-6 pathway could reduce cardiovascular events and mortality in patients receiving maintenance dialysis.

Clazakizumab is a high-affinity humanized monoclonal antibody that targets the IL-6 ligand and inhibits downstream IL-6 function. We hypothesized that clazakizumab could reduce the occurrence of cardiovascular events in patients receiving maintenance dialysis by modulating excess IL-6-dependent inflammation. We designed a double-blind, randomized, placebo-controlled combined dose-finding (phase 2b) and cardiovascular outcome (phase 3) trial in patients receiving maintenance dialysis with serological evidence of inflammation at baseline. In the current report, we describe the results of the phase 2b component of the trial, examining the safety and efficacy of three doses of clazakizumab on markers of inflammation associated with major adverse cardiovascular events.

## Results

### Patient disposition

**Fig. 1:**
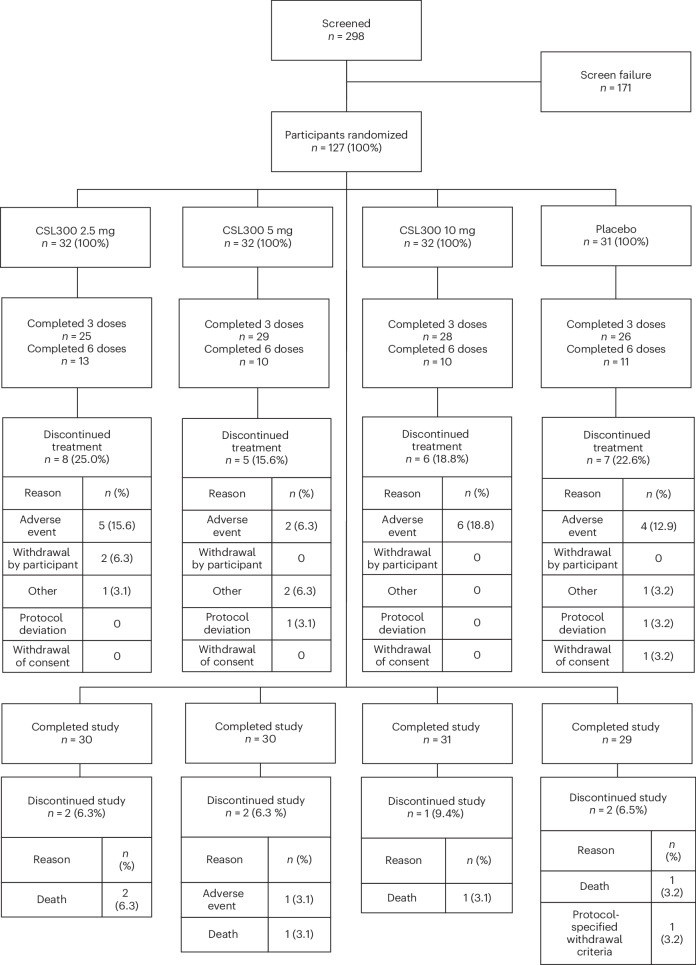
CONSORT diagram. mITT analysis included 127 participants who all received at least one dose of the study drug or placebo. Discontinued treatment refers to treatment discontinuation before the common treatment end date. CONSORT, Consolidated Standards of Reporting Trials.

Between 26 October 2022 and 17 August 2023, 298 patients provided written informed consent and underwent eligibility screening, of whom 127 were randomized (Fig. 1). Baseline demographic and clinical characteristics were generally similar across randomized groups (Table 1). The mean age was 62.4 ± 13.0 years, and 42 (33%) were women. Fifty-eight (46%) participants were designated as non-White; 36 (28%) were designated as Hispanic or Latina/Latino. The most frequently reported cause of kidney failure was diabetes (*n* = 90, 71%). Median baseline serum high-sensitivity CRP (hs-CRP) was 8.3 mg l^−1^ (25–75%, range: 4.8–19.1 mg l^−1^).

**Table 1:** Patient demographics and baseline characteristics

Demographics		Clazakizumab 2.5 mg (*n* = 32)	Clazakizumab 5 mg (*n* = 32)	Clazakizumab 10 mg (*n* = 32)	Placebo (*n* = 31)
Age (year), median (range)		63 (39, 83)	67 (36, 82)	63 (31, 83)	69 (36, 86)
Sex	Male, *n* (%)	21 (65.6)	18 (56.3)	25 (78.1)	21 (67.7)
Race	White, *n* (%)	21 (65.6)	18 (56.3)	17 (53.1)	13 (41.9)
	Black, *n* (%)	9 (28.1)	13 (40.6)	12 (37.5)	15 (48.4)
Ethnicity	Latino, *n* (%)	10 (31.3)	9 (28.1)	12 (37.5)	5 (16.1)
BMI (kg m^−^^2^), mean (s.d.)		30.5 (5.5)	30.9 (6.7)	35.1 (9.5)	32.4 (8.2)
ESKD history	Years since the first diagnosis of ESKD, mean (s.d.)	4.7 (4.5)	5.8 (4.1)	4.5 (3.2)	5.2 (3.3)
	Diabetic kidney disease, *n* (%)	21 (65.6)	25 (78.1)	26 (81.3)	18 (58.1)
	Hypertensive kidney disease, *n* (%)	8 (25)	4 (12.5)	3 (9.4)	9 (29.0)
Dialysis access	Fistula, *n* (%)	26 (81.3)	24 (75.0)	21 (65.6)	22 (71.0)
	Graft, *n* (%)	1 (3.1)	7 (21.9)	4 (12.5)	4 (12.9)
	Catheter, *n* (%)	5 (15.6)	1 (3.1)	7 (21.9)	5 (16.1)
Cardiovascular history	History of CAD	13 (40.6)	22 (68.8)	16 (50.0)	12 (38.7)
	Prior MI	5 (15.6)	2 (6.3)	5 (15.6)	3 (9.7)
	Prior PCI	2 (6.3)	6 (18.8)	2 (6.3)	3 (9.7)
	Prior CABG	3 (9.4)	5 (15.6)	4 (12.5)	3 (9.7)
	Cerebrovascular disease	3 (9.4)	3 (9.4)	6 (18.8)	6 (19.4)
	Diabetes	29 (90.6)	28 (87.5)	30 (93.8)	27 (87.1)
	Hypertension	31 (96.9)	31 (96.9)	32 (100)	30 (96.8)
	Atrial fibrillation	7 (21.9)	5 (15.6)	8 (25.0)	9 (29.0)
	Former or current use of tobacco products	8 (25.0)	11 (34.4)	14 (43.8)	13 (41.9)
Baseline medications	Statins	19 (59.4)	23 (71.9)	19 (59.4)	20 (64.5)
	Antiplatelet agents	12 (37.5)	20 (62.5)	18 (56.3)	13 (41.9)
	ESA use	22 (68.8)	24 (75.0)	23 (71.9)	22 (71.0)
	Parenteral iron	23 (78.1)	18 (56.3)	26 (81.3)	21 (67.7)
Baseline laboratory	Baseline IL-6 (ng l^−1^), mean (s.d.)	9.4 (8.2)	9.0 (8.0)	9.7 (8.6)	8.3 (5.6)
	Baseline hs-CRP (mg ml^−1^) median (25–75%)	9.5 (5.2, 32.3)	7.2 (3.6, 15.0)	8.94 (5.4, 22.9)	7.3 (4.5, 16.2)
	Serum amyloid A (mg l^−1^), median (25–75%)	10.2 (4.8, 24.3)	13.3 (8.8, 30.4)	11.8 (5.6, 29.1)	8.8 (5.6, 16.3)
	Phospholipase A2 mass (μg l^−1^), median (25–75%)	25.1 (10.3, 41.1)	26.0 (14.9, 41.7)	20.2 (12.7, 43.1)	26.2 (16.2, 42.9)
	Fibrinogen (g l^−1^), median (25–75%)	4.0 (3.3, 4.8)	4.2 (3.7, 4.8)	4.2 (3.5, 5.0)	4.0 (3.3, 4.5)
	Lipoprotein(a) (nmol l^−1^), median (25–75%)	36.7 (11.0, 94.9)	77.4 (32.5, 175.8)	55.5 (20.1, 150.7)	59.6 (22.9, 97.3)
	Albumin (g dl^−1^), mean (s.d.)	3.8 (0.21)	3.7 (0.24)	3.8 (0.25)	3.8 (0.27)

Patient disposition is illustrated in Fig. 1. The per protocol (PP) analysis set consisted of 123 patients who did not have an intercurrent event before week 12; 101 of those patients had data at week 12. The modified intention-to-treat (mITT) analysis set consisted of all 127 patients who received at least one dose of the study drug or placebo; 107 of those patients had data at week 12.

### Primary outcome

#### hs-CRP

**Fig. 2:**
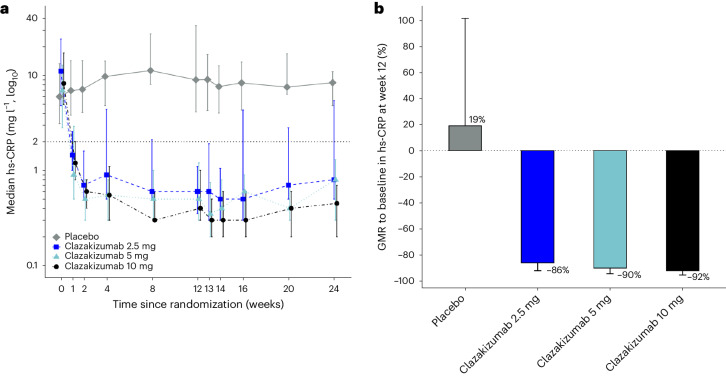
hs-CRP concentrations in the placebo and clazakizumab groups. **a**, Median (25–75%) serum hs-CRP concentrations in the placebo and three clazakizumab dose groups from baseline to week 24. The number of patients at weeks 0, 12 and 24, respectively, are placebo: 30, 26 and 10; clazakizumab 2.5 mg: 31, 24 and 13; clazakizumab 5 mg: 31, 26 and 10; clazakizumab 10 mg: 31, 25 and 10. **b**, GMRs to baseline (95% CI) of serum hs-CRP concentrations in the placebo and three clazakizumab dose groups and at week 12 after randomization are shown (*P* < 0.001 for each of the clazakizumab dose groups versus placebo). Two-sided *P* values shown are from the MMRM analysis, using a *t* test, and are not adjusted for multiplicity. Number of patients are placebo (*n* = 26); 2.5 mg (*n* = 24); 5 mg (*n* = 26); 10 mg (*n* = 25). CI, confidence interval.

**Extended Data Fig. 1:**
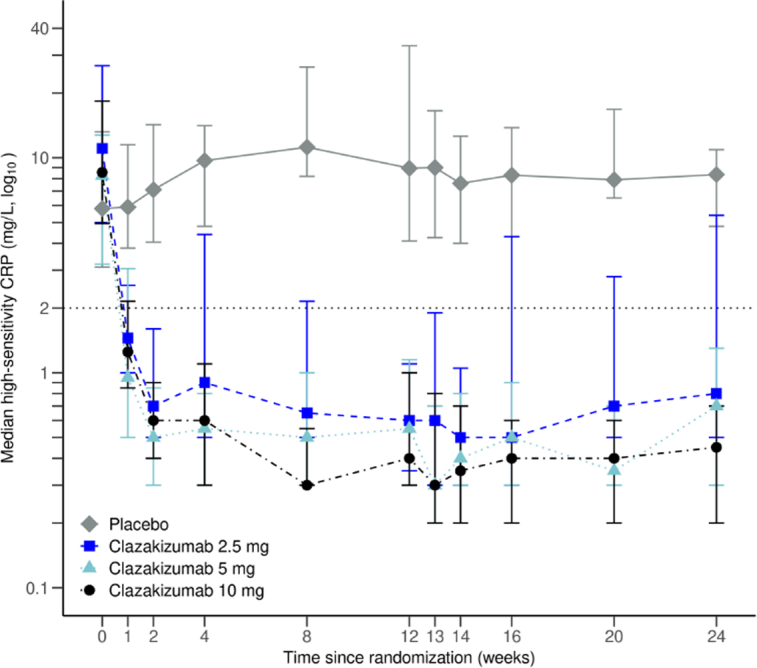
hs-CRP concentrations in the placebo and clazakizumab, mITT analysis.

All clazakizumab-treated groups experienced a rapid decline in serum hs-CRP concentrations that were maintained throughout 24 weeks of follow-up (Fig. 2a). Serum hs-CRP concentrations at week 12 decreased by 86%, 90% and 92% in patients randomized to 2.5 mg, 5 mg or 10 mg clazakizumab, respectively, and increased by 19% in patients randomized to placebo (all *P* < 0.001 compared with placebo; Fig. 2b). Corresponding values in the mITT analysis were −86%, −90%, −91% and 20%, respectively (all *P* < 0.001 compared with placebo; Extended Data Fig. 1).

**Fig. 3:**
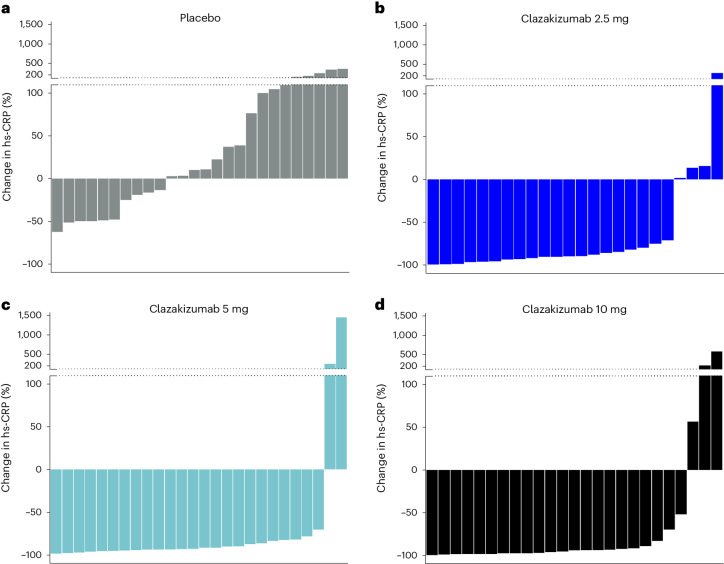
Waterfall plots of change in hs-CRP. Waterfall plots of hs-CRP percentage changes from baseline to 12 weeks of individual participants in placebo (**a**) and the three clazakizumab dose groups (mITT analysis) 2.5 mg (**b**), 5 (**c**), 10 mg (**d**).

Figure 3 shows the individual patient change in hs-CRP (waterfall plots) for all groups, illustrating a reduction in hs-CRP for nearly all patients treated with clazakizumab. In the mITT analysis, the proportion of patients who achieved hs-CRP < 2.0 mg l^−1^ was 79%, 82% and 79% in the 2.5 mg, 5 mg and 10 mg clazakizumab groups, respectively, compared with 0% of placebo-treated patients.

### Secondary outcomes: downstream biomarkers of IL-6 activity

**Fig. 4:**
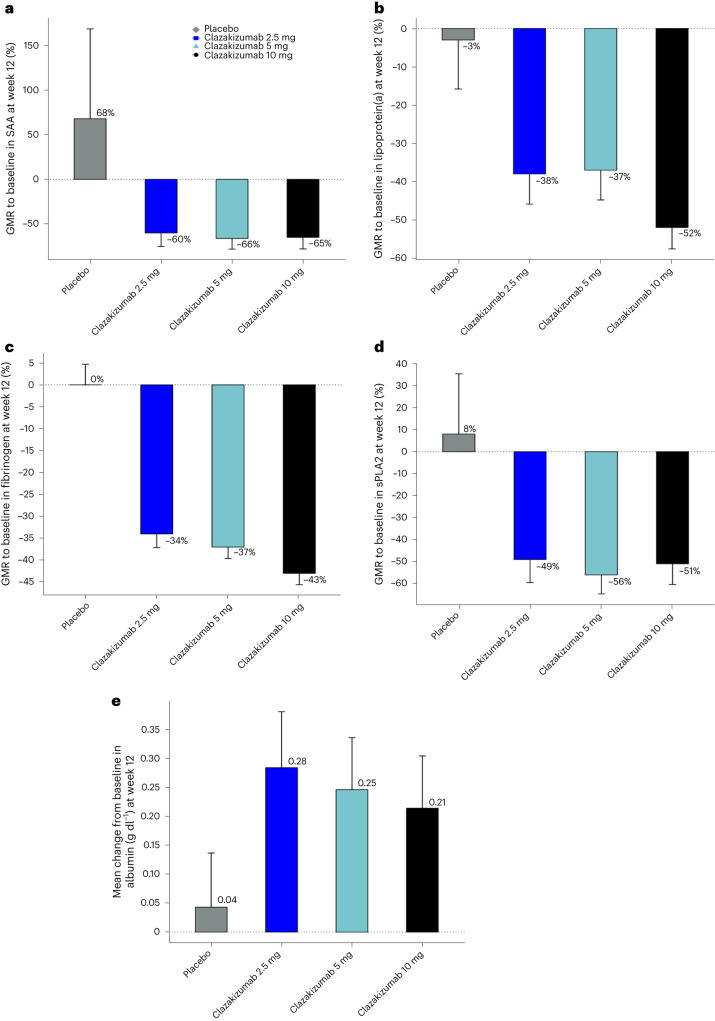
Changes in biomarkers of IL-6 activity. Changes from baseline to 12 weeks of treatment for biomarkers of IL-6 activity (mITT analysis). GMR to baseline serum amyloid A (**a**), lipoprotein(a) (**b**), fibrinogen (**c**), secretory phospholipase A2 (**d**) and albumin (**e**) (95% CI). Number of patients ranged from *n* = 25–26 for placebo, *n* = 24 for 2.5 mg, *n* = 27–28 for 5 mg and 26–29 for 10 mg.

Figure 4 shows the geometric mean ratio (GMR) to baseline in the downstream biomarkers of IL-6 activity—serum amyloid A, lipoprotein(a), fibrinogen and secretory phospholipase A2, respectively (all *P* < 0.001 compared with placebo). The mean increase in serum albumin was 0.28, 0.25 and 0.21 g dl^−1^ at 12 weeks relative to baseline in the 2.5, 5 and 10 mg clazakizumab groups, respectively, compared with 0.04 g dl^−1^ in the placebo group (all *P* < 0.01 compared with placebo).

### Safety

Adverse events are summarized in Table 2. Through the course of the study, there were two cases of Common Terminology Criteria for Adverse Events (CTCAE) grade 3 thrombocytopenia and two cases of grade 3 neutropenia, which resolved in 4 weeks when the drug was stopped. There was one case of grade 4 hypertriglyceridemia in a patient with a known history of the same. Extended Data Table 1 shows standard lipid levels, liver enzyme concentrations and platelet counts at baseline and 12 weeks. There were small increases in total cholesterol in all three clazakizumab groups, consistent with an anti-inflammatory effect^20^. Serious infections occurred in four patients in the 2.5 mg group, three patients in the 5 mg group, nine patients in the 10 mg group and two patients in the placebo group. These led to discontinuation of treatment in four patients, including two in the placebo group (Table 2). One additional patient in the 10 mg group experienced a serious infection during the 8-week final safety follow-up.

**Table 2:** Serious adverse events and adverse events of special interest

Adverse events	2.5 mg (*n* participants (%))	5 mg (*n* participants (%))	10 mg (*n* participants (%))	Placebo (*n* participants (%))
Death	2 (6.3)^a^	2 (6.3)^a^	1 (3.1)	1 (3.2)
Death related to study drug	0	0	0	0
Serious adverse event	9 (28.1)	13 (40.6)	16 (50)	7 (22.6)
Serious adverse event leading to discontinuation of study drug	5 (15.6)	2 (6.3)	6 (18.8)	4 (12.9)
Serious adverse events related to study drug	1 (3.1)	0	1 (3.1)	1 (3.2)
Serious Infections	4 (12.5)	3 (9.4)	9 (28.1)	2 (6.5)
Infection leading to discontinuation of study drug	2 (6.3)	0	4 (12.5)	1 (3.2)
Infection related to study drug	0	1 (3.1)	1 (3.1)	2 (6.5)
Infection by maximum severity				
Mild	2 (6.3)	4 (12.5)	3 (9.4)	3 (9.7)
Moderate	6 (18.8)	2 (6.3)	4 (12.5)	3 (9.7)
Severe	2 (6.3)	2 (6.3)	6 (18.8)	2 (6.5)
Hematology abnormalities^b^				
Neutropenia: CTCAE grade 3 or 4	0	2 (6.5)	0	0
Thrombocytopenia: CTCAE grade 3 or 4	1 (3.1)	0	1 (3.1)	0

Serious Infections included pneumonia, sepsis, septic shock (placebo); COVID-19, sepsis, herpes zoster oticus, endocarditis (2.5 mg); osteomyelitis, groin abscess, tooth abscess (5 mg); osteomyelitis, COVID-19, device related-infection and bacteremia, pneumonia, sepsis (10 mg).

^a^One death in this group occurred after all six doses of the study drug.

^b^Resolved with study drug interruption.

## Discussion

In this randomized, placebo-controlled, dose-finding study in patients receiving dialysis with hs-CRP ≥ 2 mg l^−1^, targeting the IL-6 pathway with clazakizumab given by intravenous bolus every 4 weeks significantly reduced serum concentrations of hs-CRP by 90% or more. Clazakizumab reduced serum concentrations of hs-CRP to <2 mg l^−1^ in approximately 80% of patients compared with 0% of patients treated with placebo. The drug was well tolerated overall with no cases of sustained moderate-to-severe thrombocytopenia or neutropenia. The proportion of patients who experienced a serious infection was similar among patients randomized to placebo, clazakizumab 2.5 mg and clazakizumab 5 mg. Serious infections were numerically more frequent among patients randomized to the clazakizumab 10 mg dose.

Chronic inflammation predicts cardiovascular events in the general population as well as in patients with dialysis- and nondialysis-requiring chronic kidney disease (CKD). In 1974, Lindner et al. published the first description of accelerated atherosclerosis in patients receiving maintenance dialysis^17^. Since then, large registry-based and other studies have highlighted the exceptionally high rates of cardiovascular events and mortality experienced by this population. Conventional therapies to lower cardiovascular risk, including lipid-lowering with statins, have shown little to no benefit in patients receiving dialysis^3–5,21^; moreover, the effects of intensification of dialysis dose—whether applied in the hemodialysis or peritoneal dialysis setting—have proved burdensome and largely unsuccessful. Multiple reports have supported the hypothesis that inflammation may substantially contribute to cardiovascular risk^22,23^. Clinical trials of anti-inflammatory agents including canakinumab, a monoclonal antibody targeting IL-1β (ref. ^12^) and colchicine^24,25^, which largely included participants with normal or near-normal kidney function, yielded sizeable reductions in the risk of cardiovascular events. IL-6 is a central inflammatory cytokine that is elevated in patients undergoing dialysis and is an independent predictor of cardiovascular events. Mendelian randomization studies have also supported a potential causal link between the activation of IL-6 and cardiovascular events^10,26–28^. In the CANTOS trial, the treatment effect of canakinumab appeared to be mediated by modulation of IL-6 levels, a downstream cytokine of IL-1. Patients with estimated glomerular filtration rate (eGFR) < 60 ml min^−1^ 1.73 m^−2^ experienced an 18% reduction in the relative risk of the primary composite endpoint of cardiovascular death or nonfatal myocardial infarction or stroke^22,29^ patients who achieved hs-CRP < 2 mg l^−1^ experienced a 32% lower risk^30^. In this phase 2b trial, approximately 80% of clazakizumab-treated patients achieved hs-CRP < 2 mg l^−1^, suggesting the potential for cardiovascular benefits with longer-term clazakizumab treatment. We also observed reductions in downstream biomarkers of IL-6 activity including a significant reduction in lipoprotein(a), which is a potential target for CV event reduction. In addition, a noteworthy effect of clazakizumab was a significant increase in albumin, not only because of the strong association between low serum albumin concentrations and mortality in patients receiving dialysis^31^ but also because few other controlled interventions have demonstrated the ability to increase serum albumin in this population^32–34^.

In the setting of mild to moderate CKD, treatment with the IL-6 ligand inhibitor ziltivekimab resulted in comparable reductions in hs-CRP and several other cardiovascular biomarkers^35^. On that basis, a large-scale cardiovascular outcomes trial is being conducted among patients with known atherosclerosis, elevated hs-CRP and eGFR ≥15 and <60 ml min^−1^ 1.73 m^−2^(ref. ^36^). The Hemodialysis Novel Therapies Consortium conducted a multicenter, randomized, placebo-controlled pilot trial of anakinra, an IL-1 antagonist, in 80 patients receiving maintenance hemodialysis with hs-CRP > 2 mg l^−1^(ref. ^37^). The median decrease in hs-CRP from baseline to week 24 was 41% in the anakinra group and 6% in the placebo group, a between-group difference that was not statistically significant. Rates of serious adverse events were virtually identical in the two groups.

Clazakizumab has been studied in multiple phase 2 studies, primarily in inflammatory diseases with generally favorable preliminary results. In a randomized, double-blind, placebo-controlled, dose-ranging study in 418 patients with rheumatoid arthritis and an inadequate response to methotrexate, clazakizumab alone or in combination with methotrexate yielded substantially reduced disease activity compared to methotrexate alone; rates of serious adverse events ranged from 8.3% to 13.6% (ref. ^38^). In a randomized, double-blind, placebo-controlled, dose-ranging study in 165 patients with active psoriatic arthritis and an inadequate response to nonsteroidal anti-inflammatory drugs, the safety profile was consistent with the pharmacology of IL-6 blockade^39^. Previously completed studies used doses ranging from 1 to 640 mg, with frequency ranging from every 3 to 8 weeks apart. Given that patients receiving maintenance dialysis are at high risk of infection, we aimed to identify one or more doses that produced a robust and sustained reduction of hs-CRP while minimizing the risk of adverse effects.

The phase 2b component of our trial has several strengths. It was randomized, double-blinded, and adequately powered for its primary outcome. Participants were broadly representative of the US dialysis population in terms of distribution by age and sex. There was ample representation of Black and Latina/Latino participants and an over-representation of patients with diabetes, by design. We obtained week 12 data on 107 (80%) participants and collected longer-term data on a sizeable fraction of patients. In addition to obtaining definitive data describing the effects of clazakizumab on hs-CRP, we demonstrated significant reductions in several other downstream biomarkers of IL-6 activity.

Limitations of the trial include a modest sample size and a relatively short duration of follow-up. It is possible that adverse effects, such as infections, might become more frequent with extended exposure to clazakizumab. We did not collect data on residual kidney function, and the sample size was insufficient to reasonably conduct subgroup analyses on selected clinical parameters (for example, male or female patients, patients with or without diabetes, patients stratified by prevalent vascular access—fistula or graft or tunneled catheter), which could have confounded the effects of clazakizumab on inflammatory markers. While we intend to include patients receiving peritoneal dialysis in the phase 3 component of the trial, no such patients participated in the phase 2b component.

In sum, among patients receiving maintenance hemodialysis with documented inflammation, clazakizumab reduced inflammatory biomarkers associated with cardiovascular events and increased serum albumin. The safety profile of clazakizumab appeared acceptable and consistent with its mechanism of action.

## Methods

### Overview

The phase 2b component of POSIBIL_6_ESKD was a parallel-group, double-blind, randomized, placebo-controlled trial conducted at 56 sites in the United States, Canada, Belgium, Germany and Australia (ClinicalTrials.gov registration: NCT05485961). A global executive committee and independent data monitoring committee (IDMC) approved the protocol and reviewed progress, quality and safety during trial conduct. Ethics Committees and Institutional Review Boards affiliated with each site approved the protocol. All participants provided written informed consent.

### Study participants

Patients aged 18 years or older receiving treatment with dialysis for 12 or more weeks with a diagnosis of cardiovascular disease and/or diabetes were eligible for screening. hs-CRP ≥ 2 mg l^−1^ measured by a central laboratory was necessary for enrollment. Exclusion criteria included positive tests for human immunodeficiency virus antibody, hepatitis B surface antigen, hepatitis C or tuberculosis using an interferon-gamma release assay, or diagnosis of a clinically significant infection within 14 days of screening; laboratory abnormalities such as neutropenia, thrombocytopenia or marked elevation of liver enzymes; recent history (<90 days) of myocardial infarction, or acute decompensated heart failure, or planned major surgery including kidney transplant; history suggestive of high risk of gastrointestinal perforation; or chronic use of immunosuppressants (full list of inclusion and exclusion criteria are included in the Supplementary Information). Eligible patients were randomly allocated in a 1:1:1:1 ratio to placebo or clazakizumab at doses of 2.5 mg, 5 mg or 10 mg. Randomization was stratified by hs-CRP 2–6 mg l^−1^ or >6 mg l^−1^ at screening to minimize the possibility of an imbalance in baseline hs-CRP across groups. All participants and investigators were blinded to treatment assignment.

### Procedures

**Extended Data Fig. 2:**
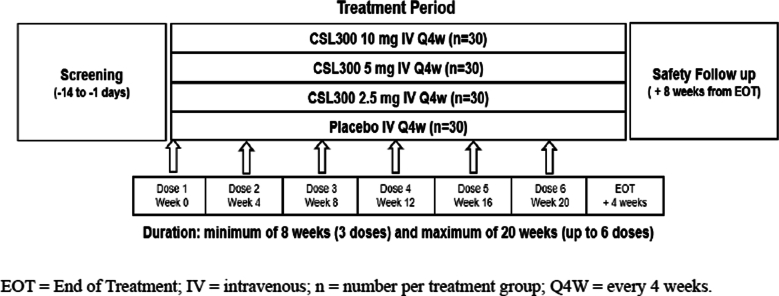
Study design schema.

Study drug, including placebo, was administered every 4 weeks as a 3-min intravenous bolus using the return venous line of the hemodialysis circuit, beginning at least 1 h after the start and no later than 1 h before completion of the dialysis session. The minimum duration of the treatment period was 12 weeks (three doses), and participants could continue to receive treatment every 4 weeks for up to six doses and followed until the last randomized participant received the third dose of the study drug, which triggered the end of the study. Thus, all patients were expected to complete the 12-week treatment period, while some patients could go on to receive treatment for up to 24 weeks to determine whether the effects were sustained longer-term and to gather additional safety information. An end-of-treatment visit occurred 4 weeks after the last dose of the study drug was received, and a safety follow-up occurred 8 weeks after (Extended Data Fig. 2).

### Efficacy outcomes

The prespecified primary efficacy outcome was the change from baseline to week 12 in serum hs-CRP, expressed as the GMR. Prespecified secondary efficacy outcomes included the following: (1) the proportion of patients who achieved hs-CRP < 2 mg l^−1^ at week 12; (2) change from baseline to week 12 in downstream biomarkers of IL-6 activity, including serum amyloid A, lipoprotein(a), fibrinogen, secretory phospholipase A2, hemoglobin, ferritin, iron, transferrin saturation and hepcidin; and (3) change from baseline to week 12 in serum albumin, a negative acute phase reactant routinely measured in patients receiving maintenance hemodialysis.

### Safety assessment

Patients were assessed for treatment-emergent adverse and serious adverse events throughout the 12- to 24-week treatment period and 8-week safety follow-up. Laboratory parameters to evaluate the safety of clazakizumab included measures of alanine aminotransferase, aspartate aminotransferase, lipid parameters and complete blood count. Laboratory measures were assessed in a central core facility (Medpace Reference Laboratories).

### Sample size

Based on experience from previous clinical trials in patients with other inflammatory diseases, we determined that a sample size of 30 participants per group (120 in total) would provide >97% power to detect an 80% reduction (GMR to placebo of 0.2, equal to −1.61 on the log scale) in hs-CRP comparing clazakizumab relative to placebo, assuming one-sided *α* of 0.025/3 to conservatively reflect multiple comparison adjustment. We assumed a s.d. of 1.4 for change from baseline on the log scale.

### Statistical analysis

The prespecified analysis data sets included a PP analysis set, mITT analysis set and safety analysis set. The mITT analysis set consists of all patients who were randomized and received any treatment. The PP analysis set consists of all patients in the mITT analysis set with no major intercurrent event potentially affecting the primary endpoint, such as death or treatment discontinuation before week 12. The safety analysis set consists of all patients who received any amount of investigational product. The prespecified primary analysis for hs-CRP used the PP analysis set to best estimate the pharmacological effect of clazakizumab, and a supplemental analysis used the mITT analysis set. Secondary endpoints were analyzed using the mITT analysis set.

To reduce variation in baseline values and mitigate misclassification, we considered the average of screening and baseline serum hs-CRP concentrations as the baseline value. For the primary efficacy outcome, we used a mixed model for repeated measures (MMRM), analyzing the change from baseline in hs-CRP on the log scale through week 24. The week 12 GMRs to placebo were expressed as the percentage difference relative to placebo. The MMRM model included terms for treatment, baseline, visit (as a categorical factor) and interactions between visit and each of the other model terms; an unstructured variance–covariance matrix was used. *T* tests from the MMRM model were used to calculate *P* values. For downstream markers of IL-6 activity, we analyzed the change (or log-transformed change for nonnormally distributed measures) from baseline using the same MMRM analysis as for hs-CRP, but including a factor for hs-CRP stratum (≤6 mg l^−1^ and >6 mg l^−1^). The primary analysis was based on the PP analysis set. Other analyses were based on the mITT analysis set. We considered two-sided *P* values < 0.05 to be statistically significant, without consideration of multiplicity. We conducted all analyses using SAS version 9.4 (SAS Institute). Results of the primary efficacy analysis were independently verified and confirmed by the Stanford Quantitative Sciences Center (Stanford University).

### Reporting summary

Further information on research design is available in the Nature Portfolio Reporting Summary linked to this article.

## Online content

Any methods, additional references, Nature Portfolio reporting summaries, source data, extended data, supplementary information, acknowledgements, peer review information; details of author contributions and competing interests; and statements of data and code availability are available at 10.1038/s41591-024-03043-1.

### Supplementary information

Supplementary Information Supplementary Note (trial organization and oversight, executive committee, steering committee, IDMC, clinical events (adjudication) committee, CSL Behring trial sponsors, institutional review boards, trial site investigators and eligibility criteria).

Reporting Summary

## Figures and Tables

**Extended Data Table 1:**
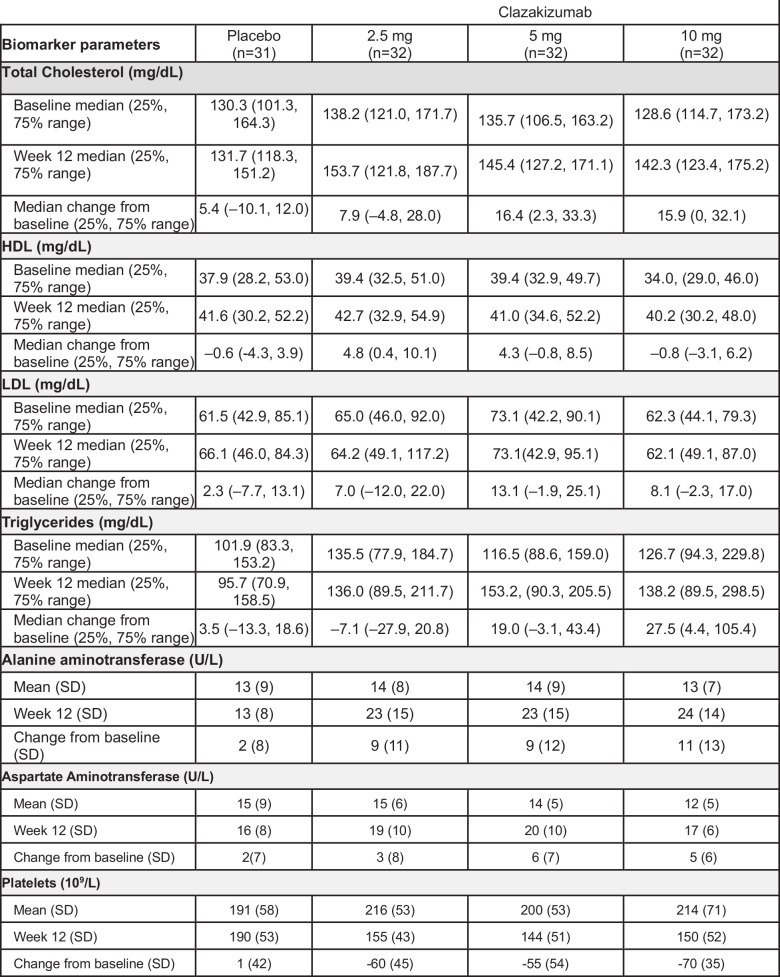
Lipid, liver enzyme and platelet parameters baseline and week 12

## Data Availability

The data that support the findings of this study are not currently available as the phase 3 study is ongoing. Individual participant data that underlie the results reported will be made available by request to the sponsor at the end of the ongoing trial, provided the proposed use of data has been approved by the review committee.
