# Author Correction: IL-6 inhibition with clazakizumab in patients receiving maintenance dialysis: a randomized phase 2b trial

**DOI:** 10.1038/s41591-024-03156-7

**Published:** 2024-07-03

**Authors:** Glenn M. Chertow, Anna Marie Chang, G. Michael Felker, Mark Heise, Elena Velkoska, Bengt Fellström, David M. Charytan, Regina Clementi, C. Michael Gibson, Shaun G. Goodman, Meg Jardine, Adeera Levin, Yuliya Lokhnygina, Jenny Mears, Roxana Mehran, Peter Stenvinkel, Angela Yee-Moon Wang, David C. Wheeler, Carmine Zoccali, Paul M. Ridker, Kenneth W. Mahaffey, Pierluigi Tricoci, Myles Wolf

**Affiliations:** 1 Stanford University, Palo Alto, CA USA; 2 CSL Behring, King of Prussia, PA USA; 3 Duke University, Durham, NC USA; 4 CSL Limited, Melbourne, Victoria Australia; 5 Uppsala University, Uppsala, Sweden; 6 New York University, New York City, NY USA; 7 Harvard Medical School, Boston, MA USA; 8 University of Toronto and University of Alberta, Edmonton, Alberta Canada; 9 University of Sydney, Sydney, New South Wales Australia; 10 University of British Columbia, Vancouver, British Columbia Canada; 11 Mt Sinai Hospital, New York City, NY USA; 12 Karolinska Institute, Solna, Sweden; 13 Hong Kong University, Hong Kong, China; 14 University College London, London, UK; 15 Ospedali Riuniti Reggio Calabria, Reggio di Calabria, Italy

**Keywords:** Vascular diseases, Kidney diseases

Correction to: *Nature Medicine* 10.1038/s41591-024-03043-1, published online 25 May 2024

In the version of the article initially published, Angela Yee-Moon Wang’s name appeared incorrectly (as Angela Wang) and has now been amended in the HTML and PDF versions of the article.

