# Supplementary material for: IL-6 inhibition with clazakizumab in patients receiving maintenance dialysis: a randomized phase 2b trial
Source: Nat Med. 2024 May 25;30(8):2328–36. doi: 10.1038/s41591-024-03043-1 (PMC11333272; doi:10.1038/s41591-024-03043-1)
Supplement: Supplementary file 1 — Supplementary Note (trial organization and oversight, executive committee, steering committee, IDMC, clinical events (adjudication) committee, CSL Behring trial sponsors, institutional review boards, trial site investigators and eligibility criteria). [file 41591_2024_3043_MOESM1_ESM.pdf]

Supplementary Materials POSIBIL$_6$ ESKD Phase2b

Trial Organization and Oversight

This trial, funded by CSL Behring (King of Prussia, Pennsylvania, USA, was collaboratively designed by members of the executive committee and the sponsor. Executive and Steering committees, chaired Glenn M. Chertow, MD and Myles Wolf, MD, are responsible for trial conduct and oversight.

Executive Committee

The Executive Committee members' affiliation are as follows.

Glenn M. Chertow, MD (Chairman), Stanford University**; Myles Wolf, MD (Study Co-Chair),** Duke Clinical Research Institute**;** David Charyatan MD, New York University, G. Michael Felker MD Duke University, Bengt Fellstrom MD, Uppsala University; Michael Gibson MD, Harvard Medical School/ Meg Jardine MD, University of Sydney; Adeera Levine MD, University of British Columbia; Yuliya Lochnigyna PhD, Duke University; Kenneth Mahaffey MD, Stanford University; Roxana Mehran, MD, Mt. Sinai Hospital; Peter Stenvinkel, MD, Karolinska University; Angela Wang, MD, Hong Kong University; Carmine Zoccali, MD, Ospedali Riuniti Reggio Calabria; Paul Ridker, MD, Harvard - Brigham & Women's; David Wheeler, MD, University College London; Shaun Goodman, MD, University of Toronto Canada. Anna Marie Chang, sponsor representative

Steering Committee

The Steering Committee is composed of the academic leadership of the clinical study, the country leaders, and members of the sponsoring company. The Steering Committee reported to the Executive Committee of the trial. The Steering Committee members' affiliation are as follows.

Rathika Krishnasamy, University of Queensland (Australia); Michel Jadoul, Cliniques Universitaires Saint-Luc (Belgium); David Collister, University of Alberta (Canada); Zuo Li, Peking University (China); My Hanna Sofia Svennson, Aalborg University Hospital (Denmark); Ziad Massy, Paris West University (UVSQ) (France); Kai-Uwe Eckhardt, Charite (Germany); Panetelis Sarafidis Aristotle, University of Thessaloniki (Greece); Andras Tisler, Semmelweis University (Hungary); Yoram Yagil Ben, Gurion University and Barzilai Medical Center (Israel); Norio Hanafusa, University Tokyo (Japan); Sunita Bavanandan, Hospital Kuala Lumpur (Malaysia); Seow Yeing Yee, Hospital Kuala Lumpur (Malaysia); Magdalena Madero Instituto Nacional de Cardiologia Ignacio Chavez (Mexico); Andrzej Wiecek, Medical University of Silesia (Katowice) (Poland); Ana Carina Costa Ferreira, Centro Hospitalar Universitario de Lisboa Central (Portugal); Ionut Nistor, Universitatea de Medicina si Farmacie (Romania); Hyeong-Cheon Park, Yonsei University (South Korea); Maria Jose Soler, University Hospital Val d'Hebron (Spain); Maiszu Wu, Taipei Medical University (Taiwan); Arkom Nongnuch Ramathibodi Hospital, Mahidol University (Thailand); Sniee Disthabanchong, Ramathibodi

Hospital, Mahidol University (Thailand); Mustafa Arici Hacettepe University (Turkey), Mark Lambie, Keele University; Simon Davies, Keele University.
Anna Marie Chang, sponsor representative

Independent Data Monitoring Committee

W. Douglas Weaver, MD (Co-Chair) Cardiology, Henry Ford Health; **Alfred K. Cheung, MD (Co-Chair) Nephrology**, University of Utah; Laura M. Dember, MD, Member Nephrology, University of Pennsylvania; Paolo Casali, MD Member Immunology, The University of Texas Long School of Medicine; Janet Wittes, PhD Member Biostatistics Wittes LLC; Cameron Wolfe, MD Member Infectious Disease, Duke University Medical Center.

Danielle Brennan, MS Independent Statistician, Non-Voting Member

Clinical Events (Adjudication) Committee:

Tara I Chang MD (Chair) Stanford University

CSL Behring – Trial Sponsor

Anna Marie Chang, MD (Clinical Research and Development, Cardiovascular and Metabolism), Pierluigi Tricoci, MD PhD (Therapeutic Area Strategy, Cardiovascular and Metabolism), Mark Heise (Director, Biostatistics), John Feaster (Associate Director, Biostatistics), Anthony Ciliberto (Senior Director, Clinical Portfolio Execution), Regina Clementi (Associate Director, Clinical Scientist), Suzanne Fink (Global Project Lead, Data Management), Christine Wong (Clinical Program Manager).

Institutional Review Boards:

(Australia) St Vincent's Hospital (Melbourne)
(Belgium) Gent, Ethics Committee, Hospital C. Heymanslaan
(USA)WCG-IRB
(Canada)University of Alberta Hospital - Health Research Ethics Board (HREB) Biomedical Panel and Queen's University and Kingston Health Sciences Centre - Health Sciences and Affiliated Teaching Hospitals Research Ethics Board (HSREB)
(Germany)Ethik-Kommission der Medizinischen Fakultät der Universität Duisburg-Essen

Trial Site Investigators

The list is in alphabetical order by investigator's last name, followed by their first name and site name, who contributed to the conduct of the POSIBIL_6 ESKD Phase2b trial.

(Australia) Kerr, P Monash Medical Centre Victoria; Pedagogos, E Sunshine Hospital Victoria; Ritchie, A Concord Hospital Concord NSW.

(Belgium) Lemahieu, W Imelda Ziekenhuis Bonheiden; Radermacher, L CHR de la Citadelle Liege; Seghers, C AZ Sint Lucas Ghent.

(Canada) Collister, D University of Alberta Hospital.

(Germany) Draganova, D Nephrologisches Zentrum; Schulte, K UKSH Klinik Innere Medizin IV

(USA) Ahmed, Jamil AKDHC Tucson Northwest Office; Ali, Slamat National Institute of Clinical Research; Assomull, Vinod Amicis Research Center; Atekha, CourageEast Georgia Research Institute; Awad, Ahmed Clinical Research Consultants, Inc; Ayodeji, Olayiwola Peninsula Kidney Associates; Bailey, Asha Columbia Nephrology; Bhat; Jodumutt Mattoo & Bhat Medical Associates, P.C.; Boiskin, Mark California Institute of Renal Research; Britton, Marcus Nephrology and Hypertension Associates, LTD; Carabello, Victor Carabello Kidney; Chowdhury, Monzurul Nephrology and Hypertension Associates PC; Christiano, Cynthia East Carolina University; Chuang, Peale Metrolina Nephrology Associates, PA; Collister, David University of Alberta Hospital; Daneshvari, Sam DaVita North Glendale; Dhillon, Kiranjit Tidewater Kidney Specialist; Echeverri, Diego South Florida Research Institute; Fadda, George California Institute of Renal Research; Fels, Eric Northeast Clinical Research Center, LLC; Gandhi, Nirav Southern California Medical Research Center; Gorlitsky, Barry DaVita Clinical Research; Hebreo, Joseph California Institute of Renal Research; Horeish, Adam California Institute of Renal Research; Hussein, Wael Satellite Healthcare; Joshi, Sucharit Seacoast Kidney and Hypertension Specialists; Joshi, Sudhir National Institute of Clinical Research; Kanade, Pushkar Great Plains Health; Kathresal, Amarnath Durham Nephrology Associates; Kooienga, Laura Colorado Kidney Care; Levine, Michael Amicis Research Center; Lynn, Robert DCR Bronx; Miller, Richard Southwest Mississippi Nephrology PLLC; Minasian, Raffi DaVita North Glendale; Navarro, Jesus Genesis Clinical Research; Newman, George Knoxville Kidney Center PLLC; Neyra, Nilda Roxana AKDHC Medical Research Services, LLC; Odren, Jennifer Center for Advanced Kidney Research, PLC; Posada, Jorge Horizon Research Group, LLC; Rubin, Oleg Amicis Research Center; Sathyan, Sharad DCR Hartford; Smith, Tamorie Renal Associates LLC; Tietjen, David Apogee Clinical Research, LLC; Tolins, Jonathan InterMed Consultants; Topf, Joel St. Clair Nephrology Research; Trespalacios, Fernando Nephrology associates of South Miami; Vera-Tapia, Edgard Kidney & Hypertension Center; Yanamadala, Sita North America Research Institute.

Eligibility Criteria

Inclusion Criteria

Subjects who met all of the following inclusion criteria could be enrolled in the study.

1. Capable of providing written informed consent by subject (or legally acceptable representative) and willing and able to adhere to all protocol requirements.
2. Male or female at least 18 years of age at the time of providing written informed consent.
3. A diagnosis of ESKD undergoing maintenance dialysis for at least 12 weeks before Screening.
4. Serum hs-CRP ≥ 2.0 mg/L measured during Screening.
5. A diagnosis of any of the following:
    a. diabetes mellitus
       OR
    b. ASCVD as evidenced by at least 1 of the following:
        i. A history of CAD, defined as at least 1 of the following:

- a history of angiography (coronary angiogram or computed tomography angiography) documenting ≥ 50% stenosis in at least 1 major epicardial vessel defined as left main coronary artery, left anterior descending artery, left circumflex artery, right coronary artery or major branches (eg, diagonal artery, obtuse marginal or posterior descending artery).
- a history of MI.
- a history of percutaneous coronary intervention (PCI) or coronary artery bypass graft (CABG).

ii. a history of PAD, defined as at least 1 of the following:

- previous revascularization procedure (eg, aortofemoral bypass surgery, axillofemoral bypass surgery, limb bypass surgery, or percutaneous transluminal angioplasty).
- previous limb or foot amputation for arterial vascular disease, or significant peripheral artery stenosis (≥ 50%) documented by angiography or by duplex ultrasound.
- a history of intermittent claudication (or equivalent ischemic symptoms) AND ankle / arm blood pressure (BP) ratio ≤ 0.9.
- previous carotid revascularization or asymptomatic carotid artery stenosis ≥ 50% diagnosed by duplex ultrasound or angiography.

Exclusion Criteria

Subjects were not enrolled into the study if they met any of the following exclusion criteria:

Exclusions Related to the Risk of Infection

1. Concomitant use of systemic immunosuppressant agents
2. Primary immunodeficiency.
3. Positive test for tuberculosis (TB) using an interferon-γ release assay (eg,QuantiFERON-TB Gold) during Screening.
4. History of latent TB without completion of full course of prophylactic treatment.
5. Evidence of HIV infection during Screening.
6. Seropositivity for hepatitis B surface antigen (HBsAg), or positive hepatitis B virus (HBV) DNA during Screening.
7. Seropositivity for hepatitis C virus (HCV) ribonucleic acid (RNA) during Screening.
8. Diagnosis of clinically significant active infection, defined as current (within 14 days of start of Screening) clinical diagnosis of infection requiring ongoing systemic antimicrobial therapy or any infection that, in the opinion of the investigator, would cause unacceptable risks to the subject if anti-IL-6 therapy is started.
9. History of OR current invasive fungal infection OR other opportunistic infection OR recurrent cellulitis (defined as 2 or more episodes in the year prior to screening).
10. Administration of a live vaccine within 6 weeks of start of Screening.
11. Presence of urinary catheter.
12. Evidence of wet gangrene or nonhealing ulcers.

Exclusions Related to Laboratory Abnormalities

13. Abnormal LFTs, ie, alanine aminotransferase (ALT), aspartate aminotransferase (AST), or total bilirubin > 2 × upper limit of normal (ULN). Subjects with a known or suspected

history of Gilbert’s syndrome are not eligible for study participation if their direct bilirubin is > 2 × ULN during Screening.

14. Neutropenia (absolute neutrophil count [ANC] < 2000/mm3).
15. Thrombocytopenia (platelet count < 100,000/mm3).

Exclusions Related to Medical History

16. Any life-threatening disease expected to result in death within 12 months (other than CVD).
17. Evidence of active hepatic disease and / or moderate or severe hepatic impairment.
18. Evidence of recent (< 90 days) MI, PCI, or CABG.
19. Evidence of decompensated heart failure as per the New York Heart Association Class III / IV clinical classifications during Screening.
20. A planned coronary revascularization (PCI or CABG) known at the time of Screening.
21. Recent (< 3 months) major surgery or planned major surgery known at the time of Screening.
22. Poorly controlled hypertension defined as predialysis BP measured consistently ≥ 170 mmHg systolic or ≥ 110 mmHg diastolic over the past month (despite use of antihypertensive therapy).
23. A present or previous (< 5 years) malignancy except for basal cell carcinoma, fully excised squamous cell carcinoma of the skin, or nonrecurrent (< 5 years of Screening) cervical carcinoma in situ.
24. Active or recent (< 30 days of Screening) clinically severe bleeding, defined as requiring transfusion of blood products for management of overt bleeding, invasive management (eg, surgical or endoscopic intervention), hospitalization for the management of bleeding, or interruption of antithrombotic treatments.
25. A scheduled kidney transplant within 6 months.
26. A history of anaphylaxis or hypersensitivity to CSL300 or any constituents of the product.
27. A history of demyelinating disorders.

Exclusions Related to the Risk of GI Perforation

28. A history of GI perforation, inflammatory bowel disease (except fully excised ulcerative colitis), or peptic ulcer disease (< 12 months before Screening).
29. A history of diverticular disease or diverticulitis (except if disease has been fully excised).
30. Inflammatory bowel disease (ie, Crohn’s disease, ulcerative colitis except if fully excised).
31. Prior gastric bypass surgery.

Exclusion Related to Treatment Compliance

32. Inadequate dialysis defined as a standard of care local value mean Kt/V < 1.2, or urea reduction ratio < 65% in the last 3 months on hemodialysis, or mean Kt/V < 1.7 on peritoneal dialysis in the last year.
33. Unwillingness or inability to comply with study procedures, including participating in another investigational study until completion of their final study visit.
34. A history of noncompliance with medical treatments including hemodialysis treatments.
35. Ongoing alcohol or illicit substance abuse.

Exclusion Related to Other Issues

36. Current or recent participation in research study involving an experimental agent < 3 months of Screening.
37. Pregnant, breastfeeding, or unwillingness to practice adequate contraception (ie, a "highly effective" acceptable method of contraception) during the study and for 5 months after the last dose of investigational product.
38. The presence of any condition that in the opinion of the investigator would
    a. compromise the safety of the subject in case of participation in the study,
    b. compromise the quality of the data, and / or
    c. limit the life expectancy of the subject to < 1 year.
39. The Sponsor determines that the subject is no longer needed for participation in study.
