# Supplementary material for: IL-6 inhibition with clazakizumab in patients receiving maintenance dialysis: a randomized phase 2b trial
Source: Nat Med. 2024 May 25;30(8):2328–36. doi: 10.1038/s41591-024-03043-1 (PMC11333272; doi:10.1038/s41591-024-03043-1)
Supplement: Supplementary file 2 — Reporting Summary [file 41591_2024_3043_MOESM2_ESM.pdf]

nature portfolio

Corresponding author(s): Glenn Chertow

Last updated by author(s): Apr 26, 2024

Reporting Summary

Nature Portfolio wishes to improve the reproducibility of the work that we publish. This form provides structure for consistency and transparency in reporting. For further information on Nature Portfolio policies, see our Editorial Policies and the Editorial Policy Checklist.

Statistics

For all statistical analyses, confirm that the following items are present in the figure legend, table legend, main text, or Methods section.

n/a	Confirmed	
☐	☒	The exact sample size (*n*) for each experimental group/condition, given as a discrete number and unit of measurement
☐	☒	A statement on whether measurements were taken from distinct samples or whether the same sample was measured repeatedly
☐	☒	The statistical test(s) used AND whether they are one- or two-sided<br>*Only common tests should be described solely by name; describe more complex techniques in the Methods section.*
☐	☒	A description of all covariates tested
☐	☒	A description of any assumptions or corrections, such as tests of normality and adjustment for multiple comparisons
☐	☒	A full description of the statistical parameters including central tendency (e.g. means) or other basic estimates (e.g. regression coefficient) AND variation (e.g. standard deviation) or associated estimates of uncertainty (e.g. confidence intervals)
☐	☒	For null hypothesis testing, the test statistic (e.g. *F*, *t*, *r*) with confidence intervals, effect sizes, degrees of freedom and *P* value noted<br>*Give P values as exact values whenever suitable.*
☒	☐	For Bayesian analysis, information on the choice of priors and Markov chain Monte Carlo settings
☒	☐	For hierarchical and complex designs, identification of the appropriate level for tests and full reporting of outcomes
☒	☐	Estimates of effect sizes (e.g. Cohen's *d*, Pearson's *r*), indicating how they were calculated

Our web collection on statistics for biologists contains articles on many of the points above.

Software and code

Policy information about availability of computer code

Data collection	All data collection occurred using iMedidata RAVE system.
Data analysis	All analyses were conducted using SAS version 9.4 (SAS Institute, Cary, NC, USA)

For manuscripts utilizing custom algorithms or software that are central to the research but not yet described in published literature, software must be made available to editors and reviewers. We strongly encourage code deposition in a community repository (e.g. GitHub). See the Nature Portfolio guidelines for submitting code & software for further information.

Data

Policy information about availability of data

All manuscripts must include a data availability statement. This statement should provide the following information, where applicable:

- Accession codes, unique identifiers, or web links for publicly available datasets
- A description of any restrictions on data availability
- For clinical datasets or third party data, please ensure that the statement adheres to our policy

We have updated the data availability statement in the manuscript to read "The data that support the findings of this study are not currently available as the Phase 3 study is ongoing. Individual participant data that underlie the results reported will be made available by request to the Sponsor at the end of the ongoing trial provided proposed use of data has been approved by review committee."

Research involving human participants, their data, or biological material

Policy information about studies with human participants or human data. See also policy information about sex, gender (identity/presentation), and sexual orientation and race, ethnicity and racism.

Reporting on sex and gender	Sex was self-reported. Gender was not considered for this study.
Reporting on race, ethnicity, or other socially relevant groupings	Race and ethnicity were based on patient's self report.
Population characteristics	Patients were adults who have evidence of inflammation (as characterized by hs-CRP >2 mg/L) with a history of diabetes or cardiovascular disease, and have been on dialysis for 12 weeks.In the "patient disposition" of the manuscript, the participants are described as "). The mean age was 62.4 ± 13.0 years and 42 (33%) were women. Fifty-eight (46%) participants were designated as non-white; 36 (28%) were designated as Hispanic or Latina/o. The most frequent reported cause of kidney failure was diabetes (n=90, 71%). Median baseline serum hs-CRP was 8.3 mg/L (25%, 75% range 4.8, 19.1 mg/L)." Additional characteristics are listed in Table 1. However, covariate adjustments were not conducted given the small sample size.
Recruitment	Patients were recruited by the site principle investigators. Potential participants were given copies of the consent form to review, and had the opportunity to take home. While there may be self-selection bias, our patient characteristics appear to represent a wide range of age, sex, and race. We involved the large dialysis organizations (Davita and Fresenius) which represent over 2/3 of the US dialysis population.
Ethics oversight	Central IRB were used for each country.<br>Australia:<br>St Vincent's Hospital (Melbourne)<br><br>Belgium:<br>Gent, Ethics Committee, Hospital C. Heymanslaan<br><br>US:<br>WCG-IRB<br><br>Canada:<br>University of Alberta Hospital - Health Research Ethics Board (HREB) Biomedical Panel<br><br>Queen's University and Kingston Health Sciences Centre - Health Sciences and Affiliated Teaching Hospitals Research Ethics Board (HSREB)<br><br>Germany:<br>Ethik-Kommission der Medizinischen Fakultät der Universität Duisburg-Essen

Note that full information on the approval of the study protocol must also be provided in the manuscript.

Field-specific reporting

Please select the one below that is the best fit for your research. If you are not sure, read the appropriate sections before making your selection.

☒ Life sciences ☐ Behavioural & social sciences ☐ Ecological, evolutionary & environmental sciences

For a reference copy of the document with all sections, see nature.com/documents/nr-reporting-summary-flat.pdf

Life sciences study design

All studies must disclose on these points even when the disclosure is negative.

Sample size	that a sample size of 30 participants per group (120 in total) would provide >97% power to detect an 80% reduction (geometric mean ratio to placebo of 0.2, equal to -1.61 on the log scale) in hs-CRP comparing clazakizumab relative to placebo, assuming one-sided alpha of 0.025/3 to conservatively reflect multiple comparison adjustment. We assumed a standard deviation of 1.4 for change from baseline on the log scale.
Data exclusions	All 127 randomized patients received at least one dose and were included in the modified Intention-to-Treat Analysis Set. The difference between the Analysis Sets is that the PP Analysis Set excluded data following intercurrent events that were likely to impact the hs-CRP results, including death prior to Week 12, missed doses or treatment discontinued, changes in background medication potentially impacting hs-CRP, kidney transplantation, or withdrawal from dialysis. In practice, only the first two of these criteria resulted in removal of data from the mITT Analysis Set to form the PP Analysis Set: 4 patients died prior to the Week 12 visit (1 from each treatment group); and individual data points from Week 12 were excluded from 6 patients who missed a dose or discontinued treatment (2 from clazakizumab 5mg group; 4 from clazakizumab 10mg group). Since only the 4 patients who died prior to Week 12 were completely excluded from the PP Analysis Set, the baseline characteristics did not differ meaningfully between the mITT and PP Analysis Sets.

Replication	Using the available data, Stanford Quantitative Sciences Unit was able to construct the same analysis set and were able to reproduce the findings.
Randomization	Eligible patients were randomly allocated in a 1:1:1:1 ratio to placebo or clazakizumab at doses of 2.5 mg, 5 mg, or 10 mg. Randomization was stratified by hs-CRP 2–6 mg/L or >6 mg/L at screening to minimize the possibility of an imbalance in baseline hs-CRP across groups.
Blinding	All participants and investigators were blinded to treatment assignment.

Reporting for specific materials, systems and methods

We require information from authors about some types of materials, experimental systems and methods used in many studies. Here, indicate whether each material, system or method listed is relevant to your study. If you are not sure if a list item applies to your research, read the appropriate section before selecting a response.

Materials & experimental systems

n/a	Involved in the study	
☒	☐	Antibodies
☒	☐	Eukaryotic cell lines
☒	☐	Palaeontology and archaeology
☒	☐	Animals and other organisms
☐	☒	Clinical data
☒	☐	Dual use research of concern
☒	☐	Plants

Methods

n/a	Involved in the study	
☒	☐	ChIP-seq
☒	☐	Flow cytometry
☒	☐	MRI-based neuroimaging

Clinical data

Policy information about clinical studies

All manuscripts should comply with the ICMJE guidelines for publication of clinical research and a completed CONSORT checklist must be included with all submissions.

Clinical trial registration	NCT05485961
Study protocol	The full protocol is not available as the Phase 3 portion is ongoing
Data collection	The study was conducted in the United States, Canada, Belgium, and Australia. Subject first visits occurred between 26 October 2022 and 17 August 2023. Last patient last visit occurred 12 Jan 2024 and database lock occurred on 21 February 2024.
Outcomes	The primary efficacy outcome was the change from baseline to week 12 in serum hs-CRP, expressed as the geometric mean ratio. Secondary efficacy outcomes included: 1) the proportion of patients who achieved hs-CRP <2 mg/L at week 12; 2) change from baseline to week 12 in downstream biomarkers of IL-6 activity, including serum amyloid A, lipoprotein(a), fibrinogen, secretory phospholipase A2, hemoglobin, ferritin, iron, transferrin saturation, and hepcidin; and 3) change from baseline to week 12 in serum albumin.
